# The use of artificial intelligence in a military context: development of the attitudes toward AI in defense (AAID) scale

**DOI:** 10.3389/fpsyg.2023.1164810

**Published:** 2023-05-05

**Authors:** Lee Hadlington, Jens Binder, Sarah Gardner, Maria Karanika-Murray, Sarah Knight

**Affiliations:** ^1^Department of Psychology, Nottingham Trent University, Nottingham, United Kingdom; ^2^School of Business, University of Leicester, Leicester, United Kingdom; ^3^Exploration Division, Defense Science and Technology Laboratory, Salisbury, United Kingdom

**Keywords:** artificial intelligence (AI), defense, scale development, attitudes, psychometric scale development and validation

## Abstract

**Introduction:**

The use of artificial intelligence (AI) for national defense is a matter of high societal significance and ongoing public discourse, but very little is known about public acceptance of AI in defense contexts. Currently, there is no reliable and valid measure of attitudes towards AI in defense, and more general attitudinal measures on AI use are unlikely to capture relevant perceptions and opinions. A measure was therefore developed for the assessment of Attitudes towards AI in Defense (AAID), and this work presents the initial validation of this scale.

**Methods:**

A total of 1,590 participants (aged 19-75, *M* = 45.7, *SD* = 16.1) completed a self-report questionnaire which included an initial item pool of 29 attitudinal statements related to the use of AI in defense. An additional general attitude towards AI scale was also included to assess the concurrently validity of the AAID scale. The AAID underwent initial statistical validation via exploratory factor analysis and confirmatory factor analysis to test the underlying structure of the newly developed scale.

**Results:**

Items reduction and exploratory factor analysis resulted in a final scale consisting of 15 items. A final two factor solution explained 42.52% of the variance (Factor 1 = 22.35%, Factor 2 = 20.17%). Factor 1 was termed “Positive outcomes” and reflected the potential and anticipated consequences of implementing AI in defense. Factor 2 was termed “Negative Outcomes” and reflected the potential negative outcomes for AI in defense. The scale also exhibited acceptable internal reliability and current validity.

**Discussion:**

The newly developed AAID presents a new measurement tool which has the capacity to assess current attitudes towards AI in defense. Such work is essential if further developments in AI in defense are to continue with the support of the public. However, the work also notes that there are some key concerns and barriers that could block further developments in the area, with further work needed to explore how such anxieties are driven by narratives related to the topic.

## 1. Introduction

The use of Artificial Intelligence (AI) continues to grow exponentially, permeating throughout many aspects of society (Schepman and Rodway, 2020). A prominent example of AI systems that people engage with in everyday live, within their homes and with some awareness of interacting with an AI system, is the smart speaker. In 2021, it was estimated that over 186 million units of smart speakers were shipped worldwide, with estimates suggesting that this could increase to more than 200 million in 2022 or 2023 (Laricchia, 2021). Also for 2021, half of all households in the UK reported to have such a device (Ofcom, 2021). Other uses of AI are much more removed from the everyday experiences of people.

The development of AI has opened up a variety of potential uses for military and defense purposes (McNeish et al., 2020). Applications for AI in defense settings are potentially unlimited, and include for example logistic support, simulation, target recognition, and threat monitoring (Taddeo et al., 2021). However, this potential is yet to be realized, and research development and the use of AI in defense is in its infancy, as reflected in current strategy outlines and policies in the Western world (Defense Innovation Board, 2019; UK Ministry of Defence, 2022). Although of crucial importance to the safety and security concerns of societies, AI in defense is arguably a domain in which the formation of informed and justified attitudes is exceedingly difficult. Members of the public are faced with information in constant flux, from governmental sources, from organizations with vested interests, from their personal networks and from popular entertainment. The present work is concerned with the measurement of attitudes toward AI in a defense context, as a first step for better understanding and explaining how such attitudes are formed.

### 1.1. Challenges and concerns surrounding AI use in the context of defense

Defense-specific definitions of AI have been offered but are not frequently discussed in the research literature. For example, the Defense Innovation Board (2019; p. 8) presented a variety of definitions associated with the use of AI in Defense:

• *“An artificial system that performs tasks under varying and unpredictable circumstances without significant human oversight, or that can learn from its experience and improve performance when exposed to data sets;*
• *An artificial system developed in computer software, physical hardware, or other context that solves tasks requiring human-like perception, cognition, planning, learning, communication, or physical action;*
• *An artificial system designed to think or act like a human, including cognitive architectures and neural networks;*
• *A set of techniques, including machine learning that is designed to approximate a cognitive task;*
• *An artificial system designed to act rationally, including an intelligent software agent or embodied robot that achieves goals using perception, planning, reasoning, learning, communicating, decision-making, and acting.”*

Such descriptions indicate that, other than the specific area of application and the types uses therein, there is little difference in what qualifies as AI within and outside civilian versus defense contexts. A summative working definition for more general purposes is provided, for example, by Gillath et al. (2021):

*“Artificial Intelligence (AI) is the simulation of human intelligence processes by machines, especially computer systems. These processes include learning (the acquisition of information and rules for using the information), reasoning (using rules to reach approximate or definite conclusions), and self-correction. Particular applications of AI include expert systems, speech recognition, and machine vision. Examples of AIs include personal helpers (like Siri and Alexa), medical diagnostic aids, and self-driving vehicles” (Gillath et al., 2021, p.1).*

Given that the purpose of the present work is not to capture accurately the technicalities of AI, but to address public perceptions of it, the general working definition by Gillath et al. (2021) can serve as an adequate and sufficiently compact description to guide the investigation in the present work.

Potential uses of AI in defense have been categorized according to three types of uses: (1) Sustainment and Support; (2) Adversarial and Non-Kinetic; and (3) Adversarial and Kinetic Uses (Taddeo et al., 2021). In brief, Sustainment and Support uses of AI in defense refer to the use of AI to support “back office” functions, such as operations and logistics (Taddeo et al., 2021). Adversarial and Non-Kinetic uses of AI include applications related to cyber-defense, as well as cyber-offense capabilities. Adversarial and Kinetic refer to the application of AI systems in combat operations, such as the use of AI systems to aid the detection of “targets” and the use of Lethal Autonomous Weapon Systems (LAWS; Taddeo et al., 2021).

Whilst the technical feasibility of AI is the focus of much research and development across several defense domains (e.g., Walsh et al., 2021), concerns related to trust, morality, legality, and ethics could pose equal, if not greater, challenges to the effective deployment of AI-led capabilities (Wasilow and Thorpe, 2019; Defense Innovation Board, 2019; Galliott and Scholz, 2020; Morgan et al., 2020). A strong reservation related to the use of AI in defense contexts relates to situations where AI-controlled machines could make decisions about and/or take human lives outside of the direct control of a human operator (Morgan et al., 2020). Further concerns surround the potential for AI systems tasked with decision support to make erroneous judgments regarding potential targets that a human operator may spot (Morgan et al., 2020). The ethics and morality of using technology for such purposes will likely play a role in attitudes toward and perceptions of AI in defense.

A number of countries have started to address concerns surrounding AI systems with an openly communicated commitment toward transparent and ethical uses in defense contexts. For example, the UK Ministry of Defence (2022) in their most recent Defense Artificial Intelligence Strategy acknowledges concerns regarding fairness, bias, reliability. Fairness concerns stem from the difficulty of establishing value-driven automated decision-making and demonstrate the need of retaining an integral human element in such processes. Biases in machine learning refer to an unwanted disproportionate impact on specific groups, here mostly with reference to military personnel. Reliability refers to stable and error-free performance of AI systems. Fairness, bias and reliability are seen, in turn, to be intricately linked to issues surrounding human responsibility and accountability, on both the side of developers and operators. Responsibility and accountability need to be maintained in the context of vast amounts of complex data to process within the very short time spans implied by computing systems. The strategy further highlights the need for public trust in AI and is fully aligned with similar commitments in the wider UK National AI Strategy (UK Government, 2022). As an integral part of such strategy, outward engagement with the public is a critical activity to ensure that a commitment to ethical use of such technologies is clearly communicated by those making decisions about their use (Morgan et al., 2020).

While strategic commitments are an important element in outreach and engagement activities, it remains to be seen whether commitments hold the power to shape and influence opinions and attitudes. In democratic societies, citizens’ views often play a key role in informed decision-making and policy development, including the regulation and funding of science and technology (Simis et al., 2016). Ultimately, the public serve to exert influence on policy and legislation as they form the electorate. If the information upon which the public are basing their key concerns is incomplete, inaccurate, or biased, this could feed into misdirected funding directions and decisions regarding further development of AI. This could have a substantial impact on national defense, particularly if other countries continue forward with developments without regard for public concerns highlighted previously (Morgan et al., 2020). As a result, it is of crucial importance to understand public attitudes regarding the use of AI in defense, and the factors that underlie these views.

At present, there is no bespoke, relevant, contemporary scale that measures attitudes toward AI in defense. Creating such a scale will help to identify key trends in public attitudes and understand factors that underlie these views, as well as being able to identify potential influences on attitudes due to information bias and misinformation. This will enable, it is hoped, technology developers to consider the reputational aspects of their practice; it will help those responsible for policy to shape relevant strategies; it will support those concerned with AI implementation to communicate appropriately to both the public and the personnel affected. Therefore, the key aim of the current study is to develop a robust, reliable, and psychometrically valid scale that is capable of measuring attitudes toward AI use in defense.

### 1.2. Understanding attitudes toward AI

Given the generality of definitions of AI discussed previously, it is challenging to adequately capture AI-related attitudes and to assess their main components or dimensions. Zhang and Dafoe (2019), in a report by the Future of Humanity Institute, explored key attitudes toward AI in a nationally representative, US-based adult sample (*n* = 2,000). This research is noteworthy, as it included detailed discussion around the preferred governance of AI. In general, participants expressed mixed feelings about the development of AI, with 41% supporting the development of AI, and a smaller number of respondents (22%) opposing further development in the field. A substantial number (82%) strongly believed that robots and AI should be carefully managed. In terms of those aspects that were rated most highly in terms of governance concerns, four key themes emerged (Zhang and Dafoe, 2019, p. 4):

1. Preventing AI-assisted surveillance from violating privacy and civil liberties;

2. Preventing AI from being used to spread fake information and harmful content online;

3. Preventing AI cyber-attacks against governments, companies, organizations, and individuals; and

4. Protecting data privacy.

Zhang and Dafoe (2019) also included two questions related to how much confidence individuals had in certain stakeholders and institutions to develop AI responsibly. When asked how much confidence they had in each actor to develop AI in the best interests of the public, it was found that most actors did not attract high levels of confidence. University researchers (50%) and the U.S. Military (49%) were the two groups most trusted to develop AI, followed by technology companies, non-profit organizations, and U.S. intelligence organizations. Secondly, when asked how much confidence they would place in each actor to manage the development and use of AI, comparable results were obtained, in the sense that the majority did not express higher levels of confidence in any institution to manage AI. While the work by Zhang and Dafoe (2019) provides valuable insights into attitudes toward AI development and governance, with some reference to a defense context, it does yield an instrument or standard operationalization of attitudes.

Research engaged more explicitly with the measurement of attitudes has focused on general applications and uses of AI. Schepman and Rodway (2020) presented the development of the General Attitudes toward Artificial Intelligence Scale (GAAIS; see also Schepman and Rodway, 2022). The GAAIS comprises 16 positive (related to opportunities, benefits, and positive emotions) and 16 negative (related to concerns and negative emotions) attitudinal statements, with examples of these being “there are many beneficial applications of Artificial Intelligence” (positive statement), and “I think that artificially intelligent systems make many errors” (negative statement). For positive statements, participants saw many applications of AI as beneficial. However, where applications of AI were perceived to threaten job security or touched on complex decisions, participants were less positive (Schepman and Rodway, 2020). Among the negative attitudinal statements, participants tended to view uses of AI that impact on privacy, or where they could be used to spy on people most negatively. Aspects concerning a loss of control over decisions, the potential unethical use of AI, and use of AI for life/death decision making were further among those perceived most negatively. In terms of overall response patterns, however, most participants did not see AI as being inherently sinister (Schepman and Rodway, 2020).

Schepman and Rodway (2020) also presented two further scales, one measuring how comfortable individuals are with specific applications, and domains of application, of AI, and the other measuring individuals’ perceptions of how capable AI is. Participants were least comfortable with applications involving complex situations that require expert knowledge or social understanding such as psychological counseling or medical consultations. Respondents were most comfortable with applications that are applied to scientific endeavors, or less personally invasive tasks, such as detecting life on other planets. Further studies involving the GAAIS have been conducted to ensure confirmatory validity of the scale (Schepman and Rodway, 2022), to investigate the moral appraisal of AI decision-making (Darda et al., 2022) and to demonstrate the perceived value of automatically compiled digital archives (Liu and Moore, 2022).

The work by Schepman and Rodway (2020, 2022) is of direct relevance to understanding attitudes related to the use of AI in a defense context. The GAAIS, however, lacks the domain-specificity that is essential for an insightful use of psychometric tools. Applying measures on generalized concepts (i.e., general attitudes toward AI) to specific domains (i.e., Defense) carries the risk of neglecting any domain-specific knowledge, opinions, concerns, and so forth. This inevitably makes it more difficult, if not impossible, to assign a justified interpretation to the measurement. As an analogous example, general measures of risk propensity are sub-optimal predictors of risky behaviors, whereas the domain-specific risk-taking scale DOSPERT has successfully shown its superiority over general instruments (Blais and Weber, 2006). The present work therefore uses the GAAIS as a useful basis to develop and design a bespoke psychometric scale specifically aimed at measuring attitudes toward the use of AI in defense.

The findings by Schepman and Rodway (2020, 2022) highlight a clear difference in attitudes toward the use of AI depending on how and why it is being used. Research using the GAAIS has shown that where AI is relied on for complex decision making, or when its use might threaten the role of the human in daily activities (e.g., job security), these applications of AI are viewed more negatively. However, more impersonal applications of AI, such as data analysis, or those that serve to remove humans from mundane or dangerous activities are rated more positively. So, in the instance of AI for defense purposes, applications that may have a direct impact on humans, particularly in “life or death” situations or when critical decision making is required, could be viewed in a more negative light; whilst applications that remove humans from risk of harm, or have less critical importance, could be viewed more positively.

Further implications for scale development that can be drawn from work on the GAAIS (Schepman and Rodway, 2020) is the need to document in a balanced way both positive and negative evaluations of AI. Overall, attitudes toward AI seem to be mixed and offering competing perspectives. One factor that may prevent more consistent attitude formation and expression comes from the fact that AI is often discussed and depicted in the context of narratives. As Cave et al. (2019) note, narratives associated with AI fall into two clear categories; (i) Positive, “Hope”-based narratives, that focus on Utopian view that AI is the answer to all of human-kind’s ills; and (ii) Negative, dystopian “Fears” that relate to humans being subjugated and being turned into slaves to ever more intelligent and sentient AI. In the most part, narratives that highlight the more negative and dystopian perspectives are in the majority (Cave et al., 2019; Schepman and Rodway, 2020).

It is possible that the influence of such narratives is exacerbated within a Defense context. As noted in the beginning, the development and use of AI in this domain is not part of everyday life experiences for most people. In addition, descriptions and definitions offered in current national-level strategies and policies are very general, while, at the same time, all things military are routinely associated with set narratives, both dystopian (Hill, 2022) and heroic (Åse and Wendt, 2018) kind. As a result, it can be expected that attitudes toward AI in defense will come with distinct positive and negative dimensions, and that a domain-specific measure needs to capture both in a way that is neither tied to accurate, but general, descriptions of the current state of affairs nor to narratives that are only loosely anchored in reality.

### 1.3. Aims and objectives

As developments within the field of AI gather pace, and developers, researchers, and policy makers explore the potential of AI in defense settings, an understanding of the attitudes of the public in this domain are essential, for reasons that are inextricably linked to the ethical dimensions of AI emergence (Jobin et al., 2019; Kerr et al., 2020). Given the scarcity of research that directly explores the attitudes of the public toward AI use in defense, there is a need to develop a new, domain-specific scale. The aims for the current study can be summarized as:

• To develop and identify the dimensionality of a new psychometric scales measuring attitudes toward AI in defense.
• To evaluate the psychometric properties of this new psychometric scale, including internal reliability and construct validity against existing scales associated with general attitudes toward AI.
• To present a refined set of items that can be used to test current attitudes toward AI in defense.

In this instance, the term “defense” is intended to encapsulate the use of AI in a wide variety of applications, including (but not limited to) military, law enforcement, intelligence, and search and rescue operations.

## 2. Materials and methods

### 2.1. Participants

A total of 1,590 participants, aged between 18–75 (*M* = 45.7, SD = 16.1) completed a self-report questionnaire. The sample comprised 777 male and 800 female participants, with 13 participants preferring not to disclose their gender. 58% reported to be in employment (excluding self-employment), 42% reported an annual income of £30,000 and above. 89% self-identified as White.

### 2.2. Development of the AAID scale

As much of the development of AI in defense is currently in its infancy, item creation for the AAID used a deductive process, guided by the available evidence on general attitudes on AI use in general (Zhang and Dafoe, 2019; Schepman and Rodway, 2020), the current outlines of potential applications in a defense context (Defense Innovation Board, 2019; McNeish et al., 2020; Taddeo et al., 2021; UK Ministry of Defence, 2022), and the popular narratives surrounding AI and related technologies (Cave et al., 2018).

In a first step, key themes and example items for further adaptation were reviewed. As outlined in the introduction, there is a general grouping into positive aspects (regarding potential and promises of progress, solutions, beneficial transformations) and negative aspects (regarding concerns and apprehensions over dystopian scenarios, unintended effects, threats to humans and humanity) prominent in work so far. Given that AI in defense does not refer to a homogenous set of established technologies, it was decided not to commit to specific applications and devices in the items wording. Instead, the focus was placed on the expected or anticipated positive and negative consequences of further development and use of AI in defense, defined in general terms. This approach means that the attitudinal construct is not represented by a direct evaluation of technology (which may or may not exist in the present), but instead by the perceived balance of positive and negative outcomes.

In a second step, draft items were generated to capture such positive and negative consequences. Negative items tapped into the dystopian narratives surrounding AI use in defense, including concerns about the threats to human existence, privacy, an escalating arms race, and ethical concerns (Cave et al., 2018; Zhang and Dafoe, 2019). Positive items aligned with a more Utopian narrative, describing AI as being beneficial in certain circumstances, being used to save lives, the protection of national infrastructure, and ushering in a new era of peace (Cave et al., 2018).

Draft items were then, in a third step, reviewed by two independent reviewers with domain knowledge. Reviewers provided feedback on general suitability and on wording such that the item pool could be further refined. This process yielded a total of 29 items, with 14 positive and 15 negative statements associated with attitudes toward AI in Defense (see Table 1). Items were further checked for readability, clarity and understanding by the researchers before being finalized for use in the study.

**TABLE 1:** Initial item pool for the AAID scale.

Item number	Item wording	Valence
1	Threaten human existence	N
2	Be used to spy on us	N
3	Lead to a new arms race	N
4	Be beneficial in humanitarian crises	P
5	Be beneficial in certain circumstances	P
6	Lead to job creation	P
7	Be hacked and turned against the people it is designed to protect	N
8	Be used to speed up critical decisions	P
9	Be used to control us	N
10	Lead to the next armed conflict	N
11	Be used unethically by those in power	N
12	Be an important technological development	P
13	Usher in a new era of peace	P
14	Be used to maintain peace	P
15	Make errors leading to collateral damage	N
16	Make armed conflict a thing of the past	P
17	Lead to unforeseen consequences	N
18	Be essential for national security	P
19	Override essential human input in critical situations	N
20	Lead to a catastrophic loss of human life	N
21	Dehumanize human conflict	N
22	Raise serious ethical issues	N
23	Be morally wrong	N
24	Be subverted by terrorist organizations and used against society	N
25	Speed up mundane/monotonous tasks	P
26	Save lives	P
27	Protect frontline staff from serious harm	P
28	Identify critical threats before they emerge	P
29	Protect critical national infrastructure	P

Items are presented in the randomized order used in the survey. Items were introduced with the stem sentence “The use of AI in Defense could…”. *P* = Positive item, *N* = Negative Item.

All 29 items were included in the survey. The section of the study was introduced with the following paragraph:

“In this part of the study, we want to now focus on the use of AI in a Defense setting. These are general statements that could relate to the use of AI for Defense purposes, and are not directly based on a particular application or type of AI. For each of the statements, please choose the option that most closely represents your attitude toward it.”

This was then followed by the stem sentence: “The use of AI in Defense could.” Participants were asked to rate the extent to which they agree with the statements on a five-point Likert-scale (from 1, strongly disagree, to 5, strongly agree).

### 2.3. Additional measures

Next to the demographic information (age, gender, employment status, income, ethnicity) and the 29 preliminary items of the AAID, the General Attitudes Toward AI Scale (GAAIS; Schepman and Rodway, 2020) was included. The GAAIS is a timely and comprehensive measure of general attitudes and was included for further validation of the AAID scale.

The GAAIS is a 20-item attitude scale which probes general attitudes toward AI. Responses are measured on a five-point Likert-scale (strongly agree—strongly disagree) and includes questions such as “There are many beneficial applications of Artificial Intelligence” (positive valence) and “I shiver with discomfort when I think about future uses of Artificial Intelligence” (negative valence). The scale is split into two, 10-item sub-scales, measuring positive and negative attitudes. The scale has been previously used with a small sample (*n* = 100), but the authors reported adequate Cronbach’s Alpha of 0.88 for the positive attitudes toward AI sub-scale, and 0.83 for the negative attitudes sub-scale, indicating good internal reliability (Schepman and Rodway, 2020). For the current study, the reliability coefficients for the negative attitudes factor were ω_*h*_ = 0.86, ω_*t*_ = 0.86, and α = 0.86 and ω_*h*_ = 0.92, ω_*t*_ = 0.92, and α = 0.91 for the positive attitudes factor.^1^

### 2.4. Procedure

Participants were invited to take part in an online survey via Qualtrics Participant Panels. Participants were supplied with full details of the study and confirmed their informed consent by selecting the relevant option with the initial stages of the survey. The data collection occurred between the 5th to the 6th April, 2022. Participants were paid a small honorarium for their time; the mean time to complete the survey was 16 min (SD = 11).

All data was cleaned at the point of collection to remove participants who exhibited response acquiescence (either agree or disagreeing to all items), and participants that completed the survey too quickly or took an excessive amount of time (in comparison to a mean time to complete). There were also attentional checks built into the questionnaire, where participants were asked to respond in a direct way to items to ensure they were still engaged in the survey (e.g., “I would be grateful if you could choose the “strongly agree” option for this item”). Participants who failed this attentional check had their participation terminated.

## 3. Results

### 3.1. Analysis overview

Prior to analysis, data were screened for missing values and normality of distributions. The data contained no missing values, and skew and kurtosis values were within acceptable ranges (all < ± 2).

The factor structure of the new Attitudes Toward AI in Defense scale was examined via Exploratory Factor Analysis (EFA) and Confirmatory Factor Analysis (CFA). All analyses were conducted in R 4.1.3 (R Core Team, 2022). Prior to running the analyses, we randomly split the sample into a training (*n* = 794) and testing (*n* = 795) sub-samples of roughly equal size. A table of the descriptive attributes for each sub-sample are presented in Table 2.

**TABLE 2:** Descriptive attributes for each sub-sample.

	*Training sub-sample* *(n* = *794)*	*Testing sub-sample* *(n* = *795)*
Age (mean years)	48.38	45.54
Sex (% female)	49.75	51.71
Ethnicity (% white)	89.96	89.39
Employment status (% employed)	64.60	66.07

Whilst a two-factor solution was hypothesized based on current literature and existing similar measures, parallel analyses (Horn, 1965) was also used to explore the dimensionality of the AAID measure. Exploratory factor analysis was run using an ordinary least squares extraction method given it is robust to asymmetric item distributions and produces unbiased rotated factor loadings (Lee et al., 2012; Asún et al., 2016). An Oblimin oblique rotation was used on the assumption that the factors would be related to one another. Items were retained if they loaded 0.50 or greater onto a factor and had a communality value greater than 0.40.

Confirmatory factor analysis was conducted using the testing sub-sample to evaluate the solution obtained via the EFA. The model fit of the CFA was evaluated using the following goodness-of-fit indices and cut-off criteria: the chi-square per degree of freedom (χ2/df) ratio (less than three deemed acceptable), robust comparative fit index (CFI; greater than 0.95 acceptable), robust Tucker-Lewis Index (TLI; greater than 0.95 acceptable), robust root mean square error of approximation (RMSEA; less than 0.06 acceptable), and the standardized root mean square residual (SRMR; less than 0.08 acceptable) Cut-off criteria were taken from West et al. (2012).

Reliability of the AAID measure was assessed with McDonald’s (1999) Omega and Cronbach’s Alpha. While Cronbach’s Alpha is known as a standard in scale construction, it relies on a number of statistical assumptions that are not necessarily met, but that are avoided by Omega (Dunn et al., 2014). Both were provided to enable some comparison.

Construct validity was further tested by inspecting Average Variance Extracted values. Concurrent validity was evaluated via the association between the new AAID scale and the existing GAAIS.

### 3.2. Item reduction and exploratory factor analysis

Item reduction and exploratory factor analysis were conducted as an iterative process with the training sub-sample. Initial item suitability was assessed via examining distribution of responses within items. Items with two or more adjacent response points averaging 10% or less of the responses were judged as having frequency problems and were removed from the item pool. All items except one (Item 5) met this criterion. Item 5 was subsequently removed from the item pool. The significance of Bartlett’s test of sphericity, χ2 (378) = 20062.26, *p* < 0.001, and the size of the Kaiser–Meyer–Olkin measure of sampling adequacy (KMO = 0.95) indicated that the remaining 28 items had sufficient common variance for factor analysis (Tabachnick et al., 2007).

The parallel analysis suggested a maximum of four factors, although only the first two factors had an empirical eigenvalue greater than one (Factor 1 = 6.91, Factor 2 = 4.58, Factor 3 = 0.93, and Factor 4 = 0.28). When examining the pattern matrix of a four-factor solution, poor interpretability of factors and weak loadings (all <0.40) were observed for Factor 3 and Factor 4. Furthermore, the pattern matrix indicated poor interpretability of the solution with only two items loading onto Factor 4 and only three items loading onto Factor 3, with evidence of item cross-loading onto two or more factors. Given these observations and the conceptual debate on attitudes presented in the introduction, a two-factor solution was explored next. Interpretability of the two-factor solution was improved; however, there was still evidence of low loading items (below 0.50), cross-loading, and poor communality values (below 0.40). Subsequently several items were removed from the item pool - Item 6, Item 13, Item 16, Item, 19, and Item 21 due to low loadings, Item 23 and Item 20 due to cross-loadings, and Item 3, Item 22, Item 10, and Item 9 due to low communality values. A final two-factor solution was then obtained with acceptable pattern loadings and communality values with the remaining fifteen items.

The final two-factor solution explained 42.52% of the variance, with Factor 1 explaining 22.35% of the variance [Eigenvalue (λ) = 6.26], and Factor 2 explaining 20.17% of the variance [Eigenvalue (λ) = 5.64]. Nine items loaded onto Factor 1 and six items loaded onto Factor 2 at 0.50 of greater. Rotated factor loadings and communalities are presented in Table 3. Factor 1 termed “Positive Outcomes” reflects potential and anticipated positive consequences of implementing AI in defense, whereas Factor 2 termed “Negative Outcomes” reflects potential and anticipated negative consequences. Pearson’s correlation indicated a weak but positive association between the two factors (*r* = 0.11).

**TABLE 3:** Pattern matrix loadings and communality values.

Item	Factor 1 loadings	Factor 2 loadings	Communality
Be essential for national security	0.74		0.56
Be beneficial in humanitarian crises	0.73		0.54
Protect frontline staff from serious harm	0.73		0.53
Protect critical national infrastructure	0.73		0.52
Save lives	0.72		0.53
Identify critical threats before they emerge	0.70		0.49
Be an important technological development	0.68		0.45
Be used to speed up critical decisions	0.65		0.42
Be used to maintain peace	0.62		0.43
Be used unethically by those in power		0.74	0.54
Be hacked and turned against the people it is designed to protect		0.73	0.54
Be used to spy on us		0.72	0.51
Lead to unforeseen consequences		0.71	0.51
Be subverted by terrorist organizations and used against society		0.69	0.47
Make errors leading to collateral damage		0.67	0.48

All cross-factor loadings <0.50.

### 3.3. Confirmatory factor analysis—two factor solution

A confirmatory factor analysis of the two-factor solution was run on the testing sub-sample, using a robust maximum likelihood estimator. The model fit for the two-factor solution indicated acceptable fit: χ2 = 375.88, df = 168.00, *p* < 0.001, χ2/df = 2.24; CFI_robust_ = 0.96, TLI_robust_ = 0.95; RMSEA_robust_ = 0.05, and SRMR_robust_ = 0.05. Furthermore, the two-factor solution provided a better fit to the data than a more parsimonious, one factor solution (AICdifference_One–Factor–Two–Factor_ = 1809.09; Burnham and Anderson, 2002).

### 3.4. Validity and reliability of the AAID scale

For establishing reliability, internal consistencies were inspected using McDonald’s ω_*h*_, ω_*t*_ and Cronbach’s α. The reliability coefficients for the negative outcomes factor were ω_*h*_ = 0.80, ω_*t*_ = 0.89, and α = 0.86 and ω_*h*_ = 0.83, ω_*t*_ = 0.92, and α = 0.90 for the positive outcomes factor and were deemed acceptable.

For an exploration of validity, Average Variance Extracted (AVE) values were computed. Next to the results of the CFA already reported, AVE values indicate how much of the observed variance is captured by a construct compared to the amount that is due to measurement error (Fornell and Larcker, 1981). Values of at least 0.50 are desirable since they indicate that at least half of the variance can be attributed to the construct. The AVE values were 0.51 and 0.50 for the negative outcomes and positive outcomes factors, respectively, and were deemed acceptable.

To examine concurrent validity, the AAID factors were examined next to the factors of the GAAIS. As the GAAIS represents a broader, top-level domain of AI applications, associations between both instruments cannot be expected to be very strong. Still, some systematic association is desirable, and this should be strongest for the two positive and the two negative factors across instruments. A full SEM model was specified, whereby both factors of the AAID and the GAAIS were modeled as latent factors, therefore accounting for measurement error. General positive attitudes toward AI were associated with both positive (*b* = 0.27, β = 0.64, *p* < 0.001) and negative attitudes toward AI in defense (*b* = 0.09, β = 0.21, *p* < 0.001). General negative attitudes toward AI were also associated with both positive (*b* = 0.11, β = 0.40, *p* < 0.001) and negative attitudes toward AI in defense (*b* = 0.13, β = 0.45, *p* < 0.001). Associations among the two positive and the two negative factors were stronger than among pairings of positive and negative factors, which was taken as first evidence of concurrent validity.

### 3.5. Scale norms for age and gender sub-groups

The final scale and instructions are presented in the Appendix. Given the low intercorrelation of the two factors obtained for the AAID, it is recommended to always inspect mean scores for the two sub-scales, Positive Outcomes and Negative Outcomes, separately. Based on the representative sample, mean scores for gender and age band can be presented here to provide comparison standards for future research. Table 4 contains mean scores for ten different age groups.

**TABLE 4:** AAID norm mean scores for gender and age.

Characteristic	*N*	Mean negative outcomes	SD negative outcomes	Mean positive outcomes	SD positive outcomes
**Sex**					
Male	777	3.68	0.75	3.62	0.64
Female	800	3.75	0.72	3.58	0.72
**Age group**					
18–24 years	153	3.73	0.65	3.50	0.61
25–29 years	127	3.71	0.73	3.47	0.63
30–34 years	183	3.68	0.76	3.63	0.69
35–39 years	162	3.69	0.75	3.61	0.56
40–44 years	127	3.77	0.69	3.65	0.63
45–49 years	148	3.75	0.70	3.71	0.56
50–54 years	163	3.75	0.75	3.51	0.76
55–59 years	130	3.66	0.86	3.56	0.83
60–64 years	136	3.78	0.71	3.56	0.72
65+ years	244	3.67	0.72	3.71	0.71

## 4. Discussion

The key aim for the current study was to develop and test a robust, psychometrically valid scale to be used as a tool to measure public attitudes toward AI use in defense. This AAID scale, presented in the Appendix, addresses a current gap in the research literature and offers the possibility to capture domain-specific attitudes instead of relying on generalized instruments. The scale contains items that do not make reference to specific AI systems or fields of application and instead focus on anticipated consequences, again of a general nature. This approach means that the AAID can remain a meaningful instrument in a context of ever-evolving technology, military priorities, and corresponding adjustments to strategy on the side of governments and societies. The main purpose of the AAID is to keep the public involved in these developments. Researchers, developers, and policy makers needs to know about current and future public attitudes, to counter misinformation, to steer own approaches to AI development and implementation, and to acknowledge and consider general sentiment, which can be expected to be closely aligned with acceptance and support.

### 4.1. Use of the AAID in research

The AAID is free to use in research. The scale presented here consists of 15 items distributed over two sub-scales: Anticipated Positive Outcomes (9 items; α = 0.90; ω_h_ = 0.83, for a more conservative estimate) and Anticipated Negative Outcomes (6 items; α = 0.86; ω_h_ = 0.80). These two factors are taken to represent positive and negative attitudes, respectively. The two factors are weakly correlated with each other, which indicates that individuals can hold, to a certain extent, both positive and negative attitudes toward the use of AI in defense. This empirical finding has implications both for scale application and implementation. First, it means that next to a consideration of an overall scale score, the scores of both sub-scales should also be routinely considered for any interpretation. Second, in some contexts that are concerned with clustering respondents or identifying different attitudinal constellations, differences can be explored between four types of responses: higher levels of positive and negative outcomes anticipated; higher levels of positive and lower levels of negative outcomes; lower levels of positive and higher levels of negative outcomes; lower levels of positive and negative outcomes.

In terms of the potential applications that are covered by the scale, the items probe the consequences of AI use for defense purposes across a wide variety of situations and contexts. The focus for the scale was initially on aspects related to AI in a military defense setting. However, on reflection, the items are worded in such a way that allows the scale to probe other situations where AI may be implemented as part of a defensive network, which could include activities aligned with law enforcement, intelligence gathering, and potentially those actions involving search and rescue operations.

### 4.2. Wider implications

In the process of design and development, several characteristics of the current state of AI, in general and in defense contexts, had to be considered, and these carry further implications for theory and debate. Most importantly, our findings support the view that there is some dissociation of attitudes and perceptions from the actual state of AI use, and that this dissociation is due to a strong future orientation and all the uncertainties that come with such an orientation. The fact that respondents could endorse both positive and negative outcomes almost independent of each other can be interpreted in several ways. It may be entirely realistic to expect such a mix of outcomes, in line with what we currently know about AI use in defense. Given the generality of definitions and the indicative nature of possible uses (see, e.g., Taddeo et al., 2021), however, it is implausible to expect that a fully informed view underpins attitudes in respondents. An alternative interpretation to consider is, therefore, that respondents draw on a variety of unranked, unfiltered, and incidental sources that allow for the generation of all kinds of expectations. This alternative interpretation implies attitudes that are not strongly rooted in fact, in communication by governments or reputed news sources, or in deeper reflection.

Popular discourse, as outlined in the beginning, is shaped by narratives, and provides ample space for positive hopes and dystopian fears (Cave et al., 2018). Elements of science fiction feature strongly in this type of discourse, in movies, games, and other forms of entertainment, which has led some commentators to use labels like the Terminator syndrome (e.g., Garvey and Maskal, 2020) to describe prominent narratives. While narratives can also be very useful, as sources of inspiration and positive vision (Cave et al., 2018), they support the formation of unfounded or false expectations surrounding emergent technologies and help to consolidate such expectations. While the present work cannot estimate the influence of narratives on attitude formation and expression, it is clear that narratives play a substantial role when members of the public are prompted to consider positive and negative outcomes of AI use in a defense context.

The potential consequences of a disconnect between current official and expert communication on what can count as factual information on the one side and utopian or dystopian narratives on the other side can be wide-ranging. AI use, both for military uses and otherwise, continues to receive increasing interest and is being engaged with by individuals outside the sphere of experts in the narrow sense of developers, dedicated academics, and domain-specific commentators. Policy makers, regulatory bodies and media organizations are all processing information and shaping opinion in their own activities (Selwyn and Gallo Cordoba, 2021). The continued dominance of narrative could, for example, lead to poor regulatory practices, to a general perception that AI developers fail to fulfill the expectations set for them, and to misguided investment decisions at the level of society.

### 4.3. Current limitations and future work

Several lines of research can be proposed at this point. On a conceptual level, the role of narratives merits further attention. As outlined in the preceding section, it may well be that current attitudes toward AI in defense are predominantly shaped by narratives. Even if this can have problematic consequences, there is no possibility to control public narratives surrounding a particular concept or technology, nor would this be desirable in open societies. However, further exploration of narratives that surround a particular concept and highlighting the limitations each has could lead to better, well-rounded communication strategies.

Next to conceptual work, more research on the validation of the AAID is needed to progress the scale development process. While the overall sample size in the present work allowed for the use of substantially sized training and testing sub-sample, cross-validation will be essential to firmly establish the psychometric robustness of the scale. Related to this point, more international studies are needed to investigate the usability of the AAID in different countries, against different contexts of AI use in military settings. This also means that different language versions are desirable, and the process of translation and subsequent validation will require detailed and rigorous work. Validation, in a next step, needs to extend to further investigation of convergent and construct validity, to test whether the two factors identified, positive and negative outcomes, show different patterns of association with proximate constructs.

## 5. Conclusion

The measurement of attitudes toward AI use in defense contexts poses specific challenges and is particularly problematic given that most individuals will have limited knowledge and interest in this area. Further challenges stem from the shifting and dynamic nature of the concepts involved and the fact that AI applications are still currently in their infancy. AI in offers a wealth of opportunities to enhance current defense capabilities, and any barrier that stalls development in this field could have a significant impact on a nation’s capacity to harness the benefits of such applications. AI in defense also has the capacity to tap into a variety of contentious topics and debates that also need to be explored alongside the current attitudes toward its use, such as the ethics, morality, and the perceived risk attached to such applications. Further, uses of AI are potentially limitless, and attitudes toward AI may vary greatly across domains of experience (e.g., private homes, financial markets, healthcare). Without more dedicated and robust attitudinal measures, there is little possibility of progressing our understanding of societal support of and opposition toward AI implementation. The present work attempts to ease some of these challenges: The AAID scale is a reliable and valid measure for further research and awaits further use.

## Data availability statement

The datasets presented in this article are not readily available because data is protected via Official Level through DSTL. Requests to access the datasets should be directed to LH, lee.hadlington@ntu.ac.uk.

## Ethics statement

Ethical approval for the current study was given a favourable opinion by the Ministry of Defense Research Ethics Committee (MODREC), ref 2109/MODREC/21. All participants provided their informed consent to participate in this study.

## Author contributions

LH: conceptual design and planning, data collection, manuscript preparation, project management, and lead researcher. JB and MK-M: data collection and manuscript preparation. SG: data collection, data analysis, and manuscript preparation. SK: manuscript preparation, approval for manuscript publication, and technical partner for funder. All authors contributed to manuscript revision, read, and approved the submitted version.

## Footnotes

1 Next to Cronbach’s Alpha, reliability in the present work was also assessed using McDonald’s (1999) Omega. See Section “3.1. Analysis overview” for further information. The survey also contained additional scales relating to trust and beliefs surrounding AI. These variables were not used in scale development, and their role in a wider multi-variate framework is presented elsewhere (IN PREP), given that the space here is devoted to the AAID specifically.

## Appendix: the AAID scale and instructions

### Instructions

“The following statements focus on the use of AI in a Defense setting. These are general statements that could relate to the use of AI for Defense purposes and are not directly based on a particular application or type of AI. For each of the statements, please choose the option that most closely represents your attitude toward it.

The use of AI in Defense could…”

**Table:**

Item	Sub-scale
2 Be used to spy on us	NO
4 Be beneficial in humanitarian crises	PO
7 Be hacked and turned against the people it is designed to protect	NO
8 Be used to speed up critical decisions	PO
11 Be used unethically by those in power	NO
12 Be an important technological development	PO
14 Be used to maintain peace	PO
15 Make errors leading to collateral damage	NO
17 Lead to unforeseen consequences	NO
18 Be essential for national security	PO
24 Be subverted by terrorist organizations and used against society	NO
26 Save lives	PO
27 Protect frontline staff from serious harm	PO
29 Protect critical national infrastructure	PO
28 Identify critical threats before they emerge	PO

The order of items reflects the original randomized order within the full item pool. Response options: 1—strongly disagree; 2—disagree; 3—neither agree nor disagree; 4—agree; 5—strongly agree.
